# Anti-malarial activity of 6-(8'Z-pentadecenyl)-salicylic acid from *Viola websteri *in mice

**DOI:** 10.1186/1475-2875-8-151

**Published:** 2009-07-07

**Authors:** Ill-Min Chung, Su-Hyun Seo, Eun-Young Kang, Won-Hwan Park, Hyung-In Moon

**Affiliations:** 1 Department of Applied Life science, Kon Kuk University, Seoul, 143-701, South Korea; 2 Department of Diagnostics, College of Korean Medicine, Dongguk University, Gyeong-Ju 780-714, South Korea; 3 Cardiovascular Medical Research Center, College of Korean Medicine, Dongguk University, Gyeong-Ju 780-714, South Korea; 4 Inam Neuroscience Research Center, Wonkwang University Sanbon Medical Center, Kyunggi-Do 435-040, South Korea

## Abstract

**Background:**

Petroleum ether extracts of *Viola websteri *Hemsl (Violaceae) were reported to have anti-plasmodial activity against *Plasmodium falciparum in vitro*, with this activity being largely attributable to 6-(8'Z-pentadecenyl)-salicylic acid (6-SA).

**Methods:**

The schizontocidal activity of 6-SA on early *Plasmodium berghei *infections was evaluated in a four-day test. The possible 'repository' activity of 6-SA was assessed using the method described by Peters. The median lethal dose (LD_50_) of 6-SA, when given intraperitoneally, was also determined using uninfected ICR mice and the method of Lorke.

**Results:**

In the present study, 6-SA was found to have anti-malarial activity *in vivo*, when tested against *P. berghei *in mice. 6-SA at 5, 10 and 25 mg/kg·day exhibited a significant blood schizontocidal activity in four-day early infections, repository evaluations and established infections with a significant mean survival time comparable to that of the standard drug, chloroquine (5 mg/kg·day).

**Conclusion:**

6-SA possesses a moderate anti-malarial activity that could be exploited for malaria therapy.

## Background

**Figure 1:**
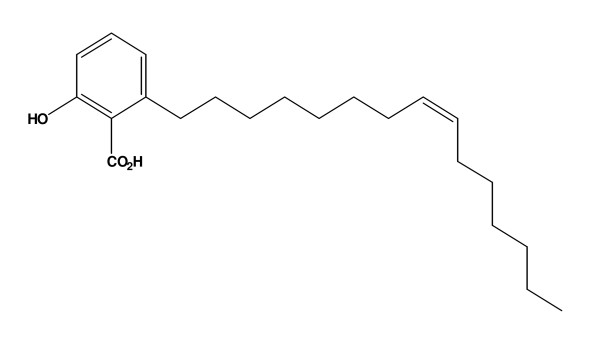
**Structure of 6-SA isolated from *Viola websteri *Hemsl**.

Malaria is a major tropical disease caused by parasites and is responsible for significant morbidity and mortality worldwide [1]. There is currently a dramatic resurgence of the disease because of the increasing resistance of the vectors to insecticides and the progressive resistance of the causative parasites, particularly *Plasmodium falciparum*, to anti-malarial drugs [2]. Therefore, there is an urgent need to discover and develop new, effective and safe drugs for the treatment of this disease [3]. Malarial parasites and plant plastids have several common pathways and functions that are fundamentally different from the analogous pathways and functions in humans and may therefore make good targets for anti-malarial drugs [4]. In traditional medicine, *Viola *is used for the treatment of insect infections, cancer, virus infection, and skin diseases [5]. A previous study [6] revealed that the petroleum ether extracts from dried whole parts of the 15 *Viola *genera screened, *Viola websteri *had inhibition values of 31.7 as percentage of parasite inhibition at 25 μg/ml. Petroleum ether extracts from *Viola websteri *were found to have anti-plasmodial activity against *P. falciparum in vitro*, with this activity being largely attributable to 6-SA (Figure 1)[7]. In the present study, 6-SA was found to have anti-malarial activity *in vivo *when tested against *Plasmodium berghei *in mice.

## Methods

### Compound preparation

Isolation of 6-SA from the plant material was reported previously [7]. The test compound was prepared by suspending the 6-SA in saline containing 0.5% Tween-80.

### Animals

As experimental hosts, 8-week-old outbred male ICR mice were purchased from Japan SLC (Hamamatsu, Japan). The animals were housed in standard cages, with standard feed and water given *ad libitum*, and they were acclimatized for 10 days prior to the experiments. All animal experiments were performed according to the guidelines for animal experimentation, Sanbon Medical Center, Wonkwang University.

### Parasite inoculation

The malarial parasite used was a chloroquine-sensitive strain of *P. berghei *(ATCC 50175; American Type Culture Collection, Manassas, VA, US), which has been maintained by serial blood passage in mice. The inoculum consisted of 5 × 10^7 ^*Plasmodium berghei *parasitized red blood cells per ml. This was prepared by determining both the percentage parasitaemia and the red blood cell count of the donor mouse and diluting the blood with isotonic saline in proportions indicated by both determinations. Each mouse was inoculated on day 0, intraperitoneally, with 0.2 ml of infected blood containing about 1 × 10^7 ^*P. berghei *parasitized red blood cell. The LD_50 _of the compound was determined using male ICR mice by intraperitoneal route using the method of Lorke *et al *[8].

### Evaluation of schizontocidal activity on early infection (4-day test)

The schizontocidal activity of 6-SA on early *P. berghei *infections was evaluated in a four-day test [9]. For this test, after determining the animal's percentage of parasitaemia and erythrocyte count, blood from an infected donor mouse was diluted with isotonic saline to obtain an inoculum containing 5 × 10^7 ^infected erythrocytes/ml. Thirty ICR mice were then (at about 07.30 hours) inoculated intraperitoneally with 0.2 ml of the inoculum before being randomly divided into six equal treatment groups of five mice each. Every morning (at 08.00–09.00 hours) from the day of infection (day 0) to 3 days later (day 3), each mouse was administered orally either 6-SA in 0.2 ml saline containing 0.5% Tween-80 (at 5, 10 and 25 mg 6-SA/kg·day), chloroquine in 0.2 ml saline containing 0.5% Tween-80 (at 5 mg chloroquine/kg·day) or 0.2 ml saline containing 0.5% Tween-80. On day 4, 24 hours after the last treatment, a thin smear was made from the tail blood of each mouse and stained with Giemsa to determine the percentage parasitaemia (by counting the number of parasitized erythrocytes per 200 erythrocytes, in random fields). For each group of mice treated with 6-SA or chloroquine, the mean percentage chemosuppression was then calculated as 100 [(A-B)/A], where A was the mean percentage parasitaemia of the mice 'treated' only with saline containing 0.5% Tween-80 (the negative controls) and B was the mean parasitaemia in the test group.

### Evaluation of the repository activity

The possible 'repository' activity of 6-SA was assessed using the method described by Peters [10]. For this, another six groups of mice (five per group) were treated pre-infection, with 0.2 ml oral doses of 6-SA in saline containing 0.5% Tween-80 (at 5, 10 or 25 mg/kg·day), pyrimethamine in saline containing 0.5% Tween-80 (at 1.2 mg/kg·day) or saline containing 0.5% Tween-80, for 4 consecutive days (days 0–3). On day 4, the mice were inoculated with *P. berghei *(as in the 4-day test) and on day 7 (72 hours post-infection) their parasitaemias were assessed.

### Evaluation of schizontocidal activity in established infection (Rane test)

To evaluate schizontocidal activity in established infection [11], the 4-day test was repeated but modified so that the first treatment did not take place until 72 hours after the mice had been infected, the mice were treated daily for five (not four) days and parasitaemias were evaluated on each day of treatment. In addition, mortality and weight changes in the mice were followed-up for 30 days post-infection (day 29) and the day-29 parasitaemias of the survivors were evaluated. The median lethal dose (LD_50_) of 6-SA, when given intraperitoneally, was also determined using uninfected ICR mice and the method of Lorke [8].

### Statistical analysis

Data were compared using Student's *t *tests, with a *P *value of < 0.05 being considered statistically significant.

## Results and Discussion

**Figure 2:**
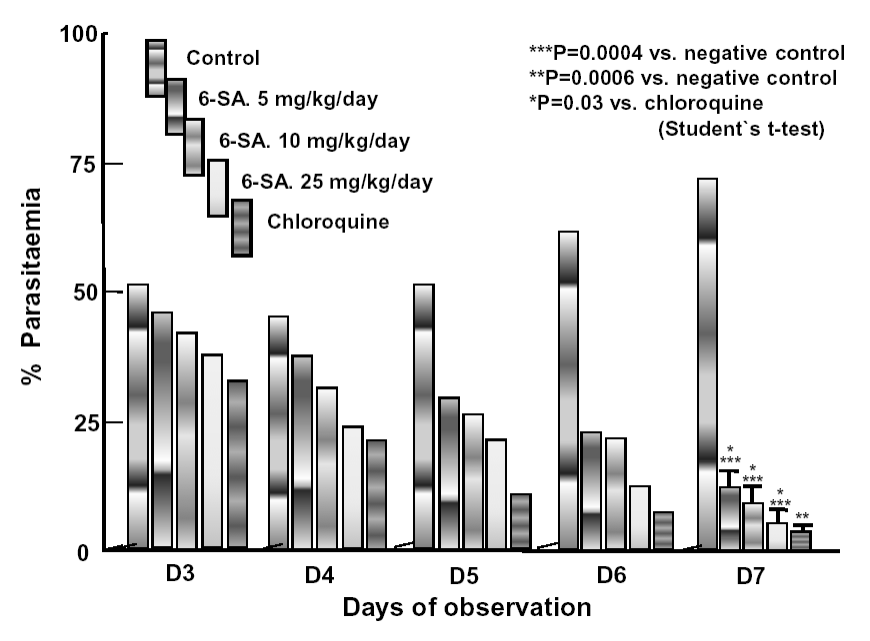
**Effect of 6-SA on established *Plasmodium berghei *infection in mice**. The experimental hosts were infected on day 0 and 'treated' orally, with saline containing 0.5% Tween-80, 6-SA at 5, 10 or 25 mg/kg·day or chloroquine at 5 mg/kg·day, on days 3–7.

In both the four-day test and the test of repository activity, oral 6-SA produced dose-dependent chemosuppression (Table 1), with even the lowest dose tested (5 mg/kg·day) causing significant reductions in parasitaemia (*P *< 0.05). The highest dose of 6-SA tested (25 mg/kg·day) did not do well enough compared with the lowest dose tested (5 mg/kg·day), and did not quite reach the level of chemosuppression seen with the drugs used as positive controls–chloroquine at 5 mg/kg·day) or pyrimethamine at 1.2 mg/kg·day (Table 1). In the mice that were treated only from 72 hours post-infection, daily oral doses of 6-SA or chloroquine led to gradual reductions in parasitaemia over time, whereas the parasitaemias in the negative-control mice increased with time (Figure 2). All the mice administered with 6-SA showed a gradual decrease in body weight from day 7, but this weight loss lasted only for a few days, after which these mice gained weight daily. Mice in the negative-control group lost weight daily during the follow-up period. One of the five mice administered with 10 mg/kg·day 6-SA died before day 29 (on day 22), as did three of the five mice treated with 5 mg/kg·day (on days 18, 19 and 25) and all five of the negative-control mice (on days 11~18). None of the mice given the highest dose of 6-SA and none of those given chloroquine died during the follow-up period. The mean survival times of the mice administered 5, 10 or 25 mg/kg·day 6-SA, chloroquine and saline containing 0.5% Tween-80 were 24.0, 27.6, 30.0, 30.0 and 15 days, respectively. The mice still alive on day 29 (all of which had been treated with 6-SA or chloroquine) were aparasitaemic. In the tests of activity against established infections, the highest tested doses of 6-SA appeared as effective as chloroquine in terms of the day-7 parasitaemias (Figure 2) and day-29 survival.

**Table 1:** Blood schizontocidal activity of 6-SA, as measured against *Plasmodium berghei *in mice

	Dose	4-day test		Repository activity	
			
Compound/Drug	(mg/kg/day)	Average % parasitaemia	Average % suppression	Average % parasitaemia	Average % suppression
6-SA	5	43.4 ± 0.25*	25.4	34.3 ± 0.37*	41.0
	10	21.5 ± 0.34*	63.0	17.4 ± 0.28*	70.1
	25	19.3 ± 0.09*	66.8	10.4 ± 0.09*	82.1
Chloroquine	5	6.4 ± 0.13*	89.0	ND	ND
Pyrimethamine	1.2	ND***	ND	4.9 ± 0.32*	91.5
Control (S.T.**)	0.2 ml	58.2 ± 0.23	-	4.9 ± 0.32*	-

* Data are expressed as mean ± for five animals per group when compared with control. *P *< 0.05.

** S.T.: saline containing 0.5% Tween-80

*** ND: Not determined.

In the toxicity tests, all the mice administered 6-SA at 5–500 mg/kg exhibited insignificant signs of toxicity, ranging from writhing and gasping (LD50 of >500 mg/kg) to decreased respiratory rate, decreased limb tone, and death. The LD50 was calculated to be >500 mg/kg. The present results indicate that 6-SA possesses useful blood schizontocidal when used at doses that cause no marked toxicity in mice. Although the mechanism of action of this compound has not been elucidated, 6-SA clearly merits further investigation.

## Conflict of interests

The authors declare that they have no competing interests.

## Authors' contributions

WHP and HIM supervised the animal studies. IMC, SHS and EYK analysed the data. All authors contributed to the drafting of the manuscript.

## References

[B1] Trager W, Jensen JB (1976). Human malaria parasites in continuous culture. Science.

[B2] Clarkson C, Maharaj VJ, Crouch NR (2004). In vitro antiplasmodial activity of medicinal plants native to or naturalised in South Africa. J Ethnopharmacol.

[B3] Biorjman A (1991). Drug resistance: changing patterns. Malaria Waiting for the vaccine.

[B4] Vella F, Ferry G, Delagrange P, Boutin JA (2005). NRH: quinone reductase 2: an enzyme of surprises and mysteries. Biochemical Pharmacol.

[B5] Lindholm P, Goransson U, Johansson S (2002). Cyclotides: a novel type of cytotoxic agents. Mol Cancer Ther.

[B6] Moon HI, Jung JC, Lee J (2007). Antiplasmodial activity of triterpenoid isolated from whole plants of Viola genus from South Korea. Parasitol Res.

[B7] Lee SJ, Park WH, Moon HI (2009). Bioassay-guided isolation of antiplasmodial anacardic acids derivatives from the whole plants of *Viola websteri *Hemsl. Parasitol Res.

[B8] Lorke D (1983). A new approach to practical acute toxicity testing. Arch Toxicol.

[B9] Knight DJ, Peters W (1980). The antimalarial activity of N-benzyloxydihydrotriazines. I. The activity of clociguanil (BRL 50216) against rodent malaria, and studies on its mode of action. Ann Trop Med Parasitol.

[B10] Peters W (1965). Drug resistance in Plasmodium berghei Vincke and Lips, 1948. I. Chloroquine resistance. Exp Parasitol.

[B11] Ryley JF, Peters W (1970). The antimalarial activity of some quinolone esters. Ann Trop Med Parasitol.

